# Supplementary material for: “It Was Just Really Hard to Be Pregnant in a Smaller Town …”: Pregnant and Parenting Teenagers’ Perspectives of Social Support in Their Rural Communities
Source: Int J Environ Res Public Health. 2022 Dec 16;19(24):16906. doi: 10.3390/ijerph192416906 (PMC9778672; doi:10.3390/ijerph192416906)
Supplement: Supplementary file 1 [file ijerph-19-16906-s001.zip › ijerph-2036301-supplementary.pdf]

Supplemental Materials

Study Interview Guide: Teenagers

Background:
Age:
Gender:
Are you pregnant or do you have any children?:

Stage-Setting – Community Context

1. How long have you lived in [X] County? Do most of your family/friends live in the area? If not, where do they live? (General geographical area)
2. How would you describe growing up in [X] County?
3. How would you describe [X] County to someone who did not grow up here?
4. What do you think is unique about [X] County? (ex: "Family First," friendly neighbors, strong faith, etc.)
5. What sort of activities were you involved in **growing up** (sports, youth-groups, boy/girl scouts, etc.) while growing up in [X] County?
    a. Can you recall any community events that were most memorable? Why?
6. Were your peers engaged in community or extracurricular activities?
    a. If not, what do you think got in the way?
    b. If so, give me examples of the community and/or extracurricular activities?
7. Reflecting on your childhood, what resources/places were available to you and your family related to staying healthy, employment, education, safety, etc.,?
    a. Any resources that stood out and made an impact on you or your family?
    b. What kinds of resources would you have liked to have had?

Adolescent Behaviors and Trends

8. How would you describe going to school in [X] County?
    a. Explain how your peers would describe going to school in [X] County

    b. When you were growing up, what types of dreams did you have about being an adult: job, family, where you would be living? *(consider coronavirus having impact on their response, feel free to address this if you feel it would be appropriate)*

    c. How has these dreams changed over time?
        1. If applicable: Has/Does becoming a teen parent impact those? How, so?

    d. What dreams do you think your parents/guardians, family, have for you as an adult--for your job, family, where you would be living?
    e. Do you have any other mentors or role models (teachers/coaches/mentor/pastor) in your life? What are their dreams for you?

    f. Tell me your thoughts about teenage pregnancy?
    g. Within your circle of friends has your group interacted with teen parents or teen pregnancy? Or within your school community?

h. Do you have classmates/friends that are/have been pregnant or are parenting teens? How did you find out/know about it?

i. How <do,did> your classmates, teachers, and family respond to teenage pregnancies?

i. Do these groups have different reactions?

9. How <do, did> teenage mothers/parents receive support while they are pregnant? (via family, peers, community)?

a. Are there organizations or businesses in town that stand out as playing a large role in supporting these mothers? What has made the most impact?

b. In what specific ways do you think community organizations can improve?

Pregnancy Process (if applicable to Teenage Parents, if not, skip to intervention prompts)

11. Would you label your pregnancy as being intentional/planned, unintentional/unplanned, or would you describe it another way?

12. Reflecting on your pregnancy experience…

a. What sorts of health and educational services were provided before, during and after your pregnancy?

b. What level of support was provided by the following people? What can be improved? What support worked well?

i. Family?
ii. School?
iii. Church (place of worship)?
iv. Father of the child?
v. Doctor/healthcare?
vi. Other community organizations?
vii. Other sources of support?

c. How many children do you have? What are their ages?

Intervention Prompts

13. Growing up, did you have conversations about sex (either via parents, community, schools)?

a. How did this education play a role in your knowledge of sexual activity?

b. Do you believe your peers had similar sexual education and thought similarly about sexual activity?

14. [X] County averages 45.3 births per 1,000 females aged 15-19 in comparison to 18.8 in the US…. What comes to mind when you hear that statistic? (*stat is from 2018, A Midwestern Youth Institute)

15. Do you believe more teenage parents need help than what is provided within [X] County?

a. If so, what organizations or groups should be involved in assisting these parents? What should be prioritized?

b. What areas of support do you think are needed most (providing teen parents with more resources, educating youth on pregnancy, educating parents on pregnancy, mental health, etc.)?

c. What do you think gets in the way of providing more support (interpersonal and organizational)?

16. What would it take to more actively support teen parents in your community?
17. What would it take to decrease unintended teen pregnancies in your community?
18. Is there anything else you would like to share that I did not ask about?

Supplemental Materials

Study Interview Guide: Adult

Background:
Age:
Gender:
Affiliation (if applicable):
Is there anyone else you would suggest recommending to participate:
- Name:
- Email/Phone:

Stage-Setting – Community Context

1. How long have you lived in [X] County? Do most of your family/friends still live in the area? If not, where do they live? (General geographical area)
2. How would you describe growing up in [X] County?
3. How would you describe [X] County to someone who did not grow up here?
4. What do you think differentiates [X] County – do you think there's a strong identity that its community members associate themselves with? (ex: "Family First," friendly neighbors, strong faith, etc.)
    a. What sort of activities were you involved in **growing up** (sports, youth-groups, boy/girl scouts, etc.) while growing up in [X] County?
        i. Can you recall any community events that were most memorable? Why?
    b. Were your peers engaged in community or extracurricular activities?
        i. If not, what do you think got in the way?
        ii. If so, give me examples of the community and/or extracurricular activities?
    c. Reflecting on your childhood, what resources were available to you and your family related to staying healthy, employment, education, safety, etc.,?
        i. Any resources that stood out and made an impact on you or your family?
        ii. What kinds of resources would you have liked to have had?

Adolescent Behaviors and Trends

5. How would you describe your experience in the [X] County schools?
    a. Do you believe your peers would share those sentiments? Why or why not?
6. Tell me your thoughts about teenage pregnancy in the [X] County community.
    a. Do you recall anyone coping with pregnancy or teen parenting while you were in school?
    b. How did peers, school administrators, and family members respond to pregnancies?
    c. Was there consensus amongst the reactions?
7. How <do, did> teen**s** receive support through their pregnancy process (via family, peers, community)?
    a. Which resources have a role in supporting these pregnant or parenting **teens**?
        i. Which of these resources have been most supportive for pregnant or parenting teens?
    b. What types of resources are missing in the [X] County community that pregnant or parenting teens need?
        i. What would it take to more actively support pregnant and teen parents in your community?

Intervention Prompts

8. Growing up, did you have conversations about sex (either with parents, friends or in schools)?
    a. More specific -- Which of this was more formal education (community-based, faith-based, school-education)?
    b. What was it like at home in terms of learning about sexual health and sexual education?
        i. Did you feel like your questions were answered?
        ii. Were there open conversations?
        iii. What was your greatest source of information?
    c. If you had any sort of sexuality education growing up how did that frame your views on sex as you got older?
    d. What were the experiences of your peers related to sexuality education?
9. Tell me about when you talked to your kid/teen about sex
    a. What prompted you to have/not have this conversation?
10. [X] County averages 32.2 births per 1,000 females aged 15-19 in comparison to 18.8 in the US…. What comes to mind when you hear that statistic?
11. What do you think priorities should be regarding teen pregnancy and teen parenting?
    a. Let's talk about a goal to develop a **teen pregnancy prevention** intervention/program in the community?
    b. What are the community priorities to prevent teenage pregnancy?
    c. What areas of support and resources do you think need to be considered when creating an intervention (providing teen parents with more resources, educating youth on pregnancy, educating parents on pregnancy, mental health, etc.)?
    d. What do you think gets in the way of providing more support (interpersonal and organizational)?
12. What would it take to reduce unintended teen pregnancies in your community?

Agency/Organization Specific Approaches

13. How has your agency/organization interfaced with teen pregnancy?
    a. Who was involved and what was the process like?
14. Explain the challenges you've had supporting teen pregnancy
15. What strategies did you use to overcome these barriers?
16. Reflecting on your work would you have approached things differently OR have things changed over time?
17. What can other organizations or departments learn from your work?
18. Are there any historical frictions amongst organizations or beneficial partnerships that have worked well or not so well in the past? How do you think we can leverage those partnerships or learn from their successes?
19. What is a common misconception about teenage pregnancy in [X] County (from community perspective, other organization, etc.)?
